# The Novel PET Radioligand [^11^C]MK‐7337 Detects Alpha‐Synuclein Pathology in Parkinson's Disease Patients

**DOI:** 10.1002/alz70856_103695

**Published:** 2025-12-26

**Authors:** Wang Yuchuan, Helen J Mitchell, Susi Lee, Zhizhen Zeng, Patricia J Miller, Idriss Bennacef, Kerry Riffel, Mona L Purcell, Hyking D Haley, Liza T Gantert, Holahan A Marie, Xiangjun Meng, Mallory Stenslik, Lei M Ma, John F Fay, Wenping Li, Jacob Marcus, Sean M Smith, Jason M. Uslaner, Robert E Drolet, Eric D. Hostetler

**Affiliations:** ^1^ Merck Sharp & Dohme LLC, West Point, PA, USA

## Abstract

**Background:**

To date, clinical feasibility of imaging Lewy‐body pathology and related disease progression in Parkinson's disease (PD) patients remains unclear. Supported by the Michael J. Fox Foundation through Ken Griffin Alpha‐Synuclein (ASYN) Imaging Award, we developed [^11^C]MK‐7337, a PET radioligand with high‐affinity to ASYN, and performed a first‐in‐human (FIH) study to determine if imaging relevant pathology in patients with mild to moderate PD was feasible.

**Method:**

Radioligand binding experiments with [^3^H]MK‐7337 were conducted in postmortem PD, Alzheimer's disease, and non‐diseased brain tissues to profile affinity and selectivity. PET imaging in the A30P (ASYN overexpressing‐transgenic) mouse model was utilized to validate the in vivo performance of tracer binding to ASYN pathology. PET imaging in pathology‐free non‐human primates (NHP) were performed to assess tracer translational suitability for PET imaging of human brains.

For the FIH study, a total of eight PD (ASYN SAA+, DAT scan+, H&Y 2‐3, on stable PD Rx) and four healthy‐elderly (HE) participants were imaged. [^11^C]MK‐7337 PET tracer uptake patterns in the brains of PD and HE participants were compared by standardized uptake value ratio (SUVR) using cortical white matter as a pseudo reference region.

**Result:**

In vitro tissue binding studies revealed MK‐7337 possessed high affinity (<1 nM) for ASYN pathology with moderate liability of off‐target binding. PET imaging in aged A30P mice demonstrated robust displaceable tracer binding in the midbrain/brainstem regions, which had known ASYN pathology. PET imaging in NHP suggested suitable tracer brain kinetics, as well as moderate off‐target binding liabilities in vivo.

The FIH study confirmed the suitable tracer kinetics of [^11^C]MK‐7337 in human brains, with peak SUV>1 in all scans. Similar patterns of tracer brain distribution and clearance were observed between HE and preclinical NHP PET scans. PD participants revealed elevated SUVRs (when compared to HE) in regions believed to contain early ASYN pathology, specifically the midbrain (including substantia nigra), brainstem/pons, and olfactory epithelium regions.

**Conclusion:**

[^11^C]MK‐7337 has demonstrated the feasibility of using a high‐affinity ASYN PET radioligand to image alpha‐synuclein pathology in PD patients.

